# Testicular vascularization in healthy dogs using a microvascular flow imaging technique - MicroV

**DOI:** 10.29374/2527-2179.bjvm004626

**Published:** 2026-09-11

**Authors:** Luisa Bittencourt de Lima Yamamoto, Diego Rodrigues Gomes, Luiz Paulo Nogueira Aires, Anna Carolina Mazeto Ercolin, Fredderico Garcia, Camila Silveira Stanquini, Desirée Rodrigues da Silva Alves, Andrea Degiovanni, Sâmara Turbay Pires, Javier Enrique Jerez Arevalo, Fernando Sebastiãn Baldi Rey, Marcus Antonio Rossi Feliciano

**Affiliations:** 1 Programa de Aprimoramento Profissional em Medicina Veterinária - Diagnóstico por Imagem, Departamento de Medicina Veterinária (ZMV), Faculdade de Zootecnia e Engenharia de Alimentos (FZEA), Universidade de São Paulo (USP), Pirassununga, SP, Brazil.; 2 Programa de Pós-Graduação em Biociência Animal (PPGBIO), ZMV, FZEA, USP, Pirassununga, SP, Brazil.; 3 Programa de Pós-Graduação em Ciências Veterinárias (Saúde Animal), Faculdade de Ciências Agrárias e Veterinárias (FCAV), Universidade Estadual Paulista “Júlio de Mesquita Filho” (UNESP), Jaboticabal, SP, Brazil.; 4 Department of Veterinary Science, University of Turin, Grugliasco, Torino, Italy; 5 Autonomous veterinarian, Pirassununga, SP, Brazil.; 6 ZMV, FZEA, USP, Pirassununga, SP, Brazil.

**Keywords:** dogs, color Doppler, microvascularization, testis, cães, Doppler colorido, microvascularização, testículo

## Abstract

Testicular health significantly impacts the overall health and reproductive capacity of dogs, making early diagnosis of testicular conditions crucial. Neoplasms, orchitis, degeneration of the seminiferous epithelium, cryptorchidism, and testicular atrophy are among the most common testicular diseases in dogs. Ultrasonography is essential in andrological examinations, as it is a safe, easy-to-perform, and minimally invasive method. However, conventional Doppler techniques are often insufficient for characterizing testicular vascularization, particularly for detecting small-caliber blood vessels and low-velocity blood flow. This study evaluated the applicability of microvascular flow imaging (MicroV) in assessing testicular microvascularization in healthy dogs. Twenty-five intact male dogs aged 1-12 years were evaluated. The examination included physical and reproductive assessments, followed by B-mode and color Doppler ultrasonography of the testes. The MicroV technique was applied to provide detailed hemodynamic information, including the visualization of diffusely branched microvessels within the parenchyma. The technique offered advantages over color Doppler in characterizing testicular vascularization and microvascularization in dogs, particularly in detecting low-flow vascular calibers, thus representing a promising complementary tool for future clinical studies on testicular abnormalities.

## Resumo

A saúde testicular impacta significativamente a saúde geral e a capacidade reprodutiva dos cães, tornando fundamental o diagnóstico precoce de afecções testiculares. Entre as doenças testiculares mais comuns em cães, estão neoplasias, orquite, degeneração do epitélio seminífero, criptorquidia e atrofia testicular. A ultrassonografia é essencial ao exame andrológico, sendo um método seguro, de fácil realização e minimamente invasivo. Entretanto, técnicas Doppler muitas vezes são insuficientes na caracterização da vascularização testicular, principalmente na determinação de vasos sanguíneos de pequeno calibre e fluxos de baixa velocidade. O presente artigo avaliou a aplicabilidade do método de imagem de fluxo microvascular (MicroV) na caracterização da microvascularização testicular em cães hígidos. Foram avaliados 25 cães machos inteiros entre um e 12 anos. O exame incluiu avaliação física e reprodutiva, seguida de ultrassonografia modo B e Doppler colorido testicular. A técnica MicroV foi aplicada para detalhamento hemodinâmico, incluindo a visibilização de microvasos difusamente ramificados pelo parênquima. MicroV ofereceu vantagens sobre a técnica de Doppler colorido, para caracterização da vascularização e microvascularização testicular em cães saudáveis, principalmente para vasos sanguíneos de baixo fluxo, tornando-se uma ferramenta complementar promissora para futuros estudos clínicos com alterações testiculares.

## Introduction

Testicular disorders are of significant importance in small animal practice, affecting the health and reproductive capacity of dogs. The investigation of such diseases, with the aim of early diagnosis, is essential to preserve animal health, the genetic quality of canine populations, and the selection of breeding stock (Domingos & Salomão, 2011). The same authors reported that among the diseases that most affect canine testes, testicular neoplasms (the second most common neoplasm in dogs), orchitis, seminiferous epithelium degeneration, cryptorchidism, and testicular atrophy stand out.

As in humans, ultrasonography is the imaging method of choice and is essential for general evaluation of the reproductive tract and for diagnosing testicular lesions in dogs. The combination of conventional ultrasonography (B-mode and Doppler) with advanced ultrasound modalities is becoming increasingly widespread. As a result, more diagnostic options are available with greater speed and accuracy in clinical and andrological practice, especially in relation to canine testicular health (Mantziaras & Luvoni, 2020). Ultrasound is recognized as the gold standard technique due to its high safety, ease of use and interpretation, and minimal invasiveness. Furthermore, it is less expensive than computed tomography and magnetic resonance imaging (Bracco et al., 2023). Thus, different ultrasonographic techniques have proven highly sensitive for the complementary diagnosis of lesions in the testicular parenchyma (Kinns & Nelson, 2019).

Doppler mode, in combination with B-mode, is frequently used for testicular evaluation, allowing the study of testicular hemodynamic vascular structure and the direction and type of blood flow (Carvalho et al., 2008). However, this technique may be limited by the animal's movement (Karaca et al., 2016), as it captures signals from both the movement of red blood cells within the vessels and that of tissues within the body. During the examination, a “wall filter” can be used to minimize motion artifacts. This mathematical algorithm removes signals unrelated to the main, high-speed blood flow. However, smaller-caliber vessels are often excluded, limiting the visualization and interpretation of the complete vascular network (Esaote, 2026; Visalli et al., 2024).

Recent microvascular flow imaging (MVFI) techniques, such as Ultrasound Microvascular Imaging (MicroV) (Esaote, Italy), can filter and separate signal acquisition and random movements, thereby reducing noise and allowing for a more sensitive and higher-resolution assessment of low-caliber vessels and low flow velocities, improving hemodynamic studies of different body systems (Aziz et al., 2022; Leong et al., 2020; Visalli et al., 2020). This new algorithm has a greater ability to select low frequencies based on their origin, preserving the image of truly weak flows and eliminating only disturbing interference (Visalli et al., 2024). Consequently, it provides a more detailed vascular map, composed of macro- and microvasculature, including main vessels and smaller subdivided lateral branches, facilitating the interpretation of normal and, consequently, pathological conditions (Visalli et al., 2024).

Besides greater sensitivity and resolution than color Doppler and power Doppler techniques, MicroV images also have an advantage over computed tomography and magnetic resonance imaging because they do not require intravenous contrast agents, which can be nephrotoxic (Aziz et al., 2022). The technique can also significantly reduce the exam duration and the need for sedation or anesthesia, as with the previously mentioned modalities (Karaca et al., 2016).

Although already widely used in human medicine, this imaging modality is still rarely used in veterinary medicine. Few studies were identified evaluating the renal parenchyma of dogs and cats. The feasibility of microvascular ultrasound for evaluating renal perfusion was described in healthy cats (Todd-Donato et al., 2025). The combined use of color Doppler and superb microvascular imaging was far more effective for assessing canine renal cortical thickness compared to B-mode alone (Koh et al., 2025). However, to our knowledge, no veterinary studies have specifically addressed the testicular microvascular evaluation in dogs. Thus, this study aimed to evaluate the applicability of microvascular flow imaging (MicroV) for characterizing testicular microvascularization in healthy dogs, as well as the method’s detection capacity and spatial resolution in small, low-flow vessels.

## Material and methods

This study was conducted at the Veterinary Hospital (HOVET) of the Faculty of Animal Science and Food Engineering of the University of São Paulo (FZEA/USP) and was previously approved by the Ethics Committee on Animal Use (CEUA) of the same institution under protocol no. 5248010623.

Twenty-five intact male dogs weighing up to 50 kg were included and originated from private or experimental kennels in the Pirassununga, São Paulo, Brazil region, as well as patients from the HOVET. The selected animals were aged 1-12 years and divided into three groups according to age: Group 1 (G1) up to 4 years; Group 2 (G2) from 4 to 8 years; and Group 3 (G3) from 8 to 12 years.

Following selection of the animals, anamnesis was performed to obtain reproductive history, general physical examination, and reproductive tract examination, comprising scrotal and testicular palpation, preputial palpation, and visual inspection of the exposed penis up to the level of the *bulbus glandis*. The presence of adhesions, signs of inflammation, nodules, or changes in consistency was considered one of the exclusion criteria.

### Color Doppler ultrasound and MicroV

To perform the testicular ultrasonographic examination, the animals were placed in dorsal recumbency on a padded support, and acoustic coupling gel was applied directly to the scrotum for subsequent scanning. No scrotal trichotomy was performed, as the scrotal region is naturally hairless or sparsely haired, thereby minimizing the risk of skin irritation, lesions, and potential allergic reactions.

The ultrasound examinations were conducted by two ultrasound technicians in the ultrasonography room, using the MyLabTM X8VET device (Esaote, Genoa, Italy) equipped with microconvex (3-11MHz) and linear multifrequency electronic transducers (4-15MHz and 4-20MHz).

Initially, the testes were evaluated in B-mode in the sagittal (longitudinal) and transverse planes to assess the testicular parenchyma. The ultrasonographic evaluation included the morphology, position, echotexture, echogenicity, and volume of the testes. Next, testicular vascularization was assessed using color Doppler mode.

Image acquisition settings, including gain adjustments and transducer selection, were adapted according to patient size and image quality requirements during the examination, following routine clinical practice. The average depth was 48mm, ranging from 28 to 78mm. Average color Doppler frequency was 5.7MHz, ranging from 5 to 10MHz. The average wall filter was 2.76, ranging from 2 to 3. Average persistence was 4.58, ranging from 3 to 6. Color Doppler gain was set at 42,3% on average, ranging from 35 to 50%. The scale/PRF was set at 1.24, on average, ranging from 0.37 to 1.9. The Doppler sampling box was adjusted to encompass the entire testicle, ensuring complete evaluation of the vascular signal within the organ.

For the assessment and hemodynamic characterization of testicular microvascularization, the microvascular flow imaging (MicroV) method of the MyLabTM X8VET device (Esaote, Genoa, Italy) was used. Color Doppler and MicroV images were acquired and simultaneously evaluated using a dual-screen display, enabling direct comparison between the two techniques under identical imaging conditions. The box size was adjusted to accommodate the whole evaluated testicle. The focal zone was maintained at the same depth in both modalities to ensure consistency in image acquisition and vascular assessment.

Furthermore, a quantitative study was performed measuring the pixel intensity in color Doppler and MicroV images, comparatively, using a digital image analysis software (ImageJ).

### Pixel quantification

**Figure 1:**
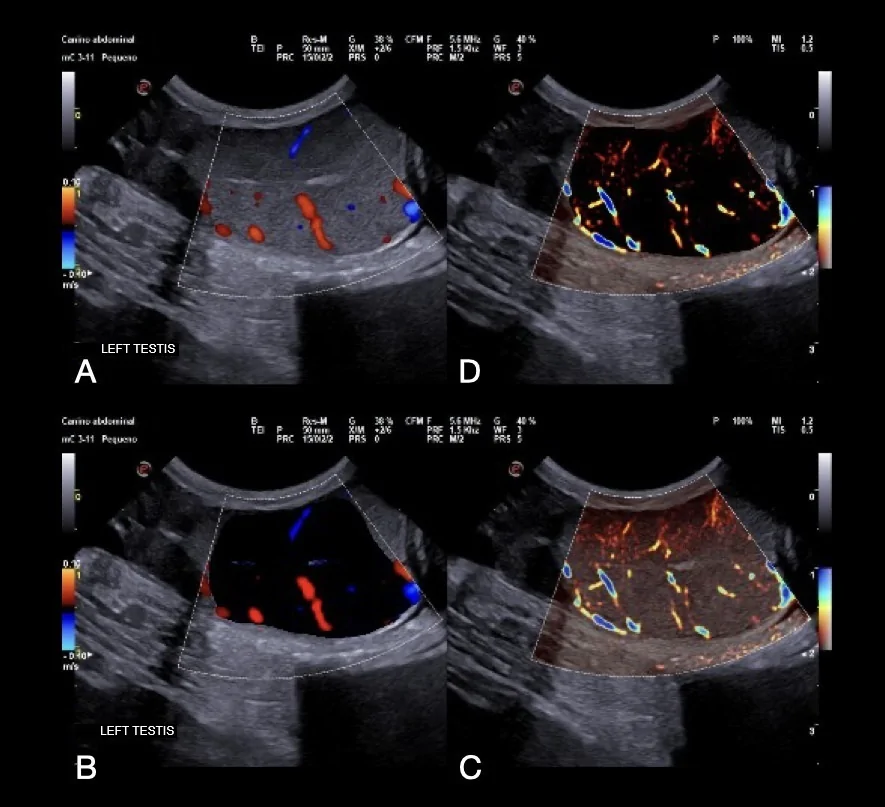
Assessment of testicular vascularization using color Doppler (A) and MicroV (C), and the corresponding images after background subtraction within the region of interest (B and D, respectively) for pixel quantification.

Image analysis was performed by a single trained and blinded operator using ImageJ/Fiji software (National Institutes of Health, Bethesda, MD, USA). Initially, the region of interest was manually delineated to exclude regions outside the field of analysis. Next, manual adjustments were made to contrast and brightness to remove background noise and isolate vascular structures (Figure 1). The operator followed a consistent processing approach for all images, adjusting contrast and brightness only to the extent necessary to obtain a homogeneous black background while preserving the integrity and continuity of the colored vascular structures. Pixel quantification was performed by obtaining the mean number of pixels (which corresponded to the total number of pixels corrected for the evaluated area. This mean was calculated automatically by the ImageJ software. The results were exported for further statistical analysis.

### Statistical analysis

The characteristics of testicular vascularization observed during the examinations were described descriptively, and a frequency distribution of these features was subsequently performed.

Data were analyzed using a split-plot design and mixed models. The number of pixels detected by ultrasonography was considered the response variable. Ultrasonographic technique was included as the main fixed effect, whereas age and body weight were included as covariates. Animal and testis nested within animal were included as random effects, in addition to the residual error, according to the model:


Yij=β0+β1(Technique)ij+β2(Age)ij+β3(Body Weight)ij+uj(Animal/Testis)+εij (1)

Where:

- Y*ij* = number of pixels detected by ultrasonography;
- *β*0 = intercept;
- *β*1, *β*2, and *β*3 = regression coefficients for the fixed effects;
- u*j*(Animal/Testis) = random effect of testis nested within animal;
- ε*ij* = residual error.

Because the response variable (number of pixels) showed a markedly asymmetric distribution, a generalized linear mixed model with a negative binomial distribution and a log link function was fitted. Fixed effects were evaluated using Type III tests. Least squares means were compared when appropriate, and statistical significance was set at P <0.05. Analyses were performed using the PROC GLIMMIX procedure of SAS (SAS Institute Inc., 2023).

## Results

Twenty-five dogs were included, all of which showed no abnormalities on physical and reproductive examination. Of these, 14 dogs were part of G1, 6 of G2, and another 5 of G3. Body weight and age of the dogs in each group were detailed in Table 1.

**Table 1:** Demographic data from the 25 evaluated dogs.

**Sample**			**Minimum**			**Maximum**	**Standard**
	**Group**	**Size**	**Mean**	**Median**	**Value**	**Value**	**Deviation**
**Bodyweight (Kg)**	G1	14	17.21	8.5	3	50	17.12
	G2	6	31.5	32	21	40	6.83
	G3	5	30	33	15	40	9.82
**Age (months)**	G1	14	19.92	18	6	36	10.62
	G2	6	51	44	36	84	18.40
	G3	5	105.6	108	96	120	10.03

For group 1, eight small-sized dogs weighing <10kg and six dogs weighing >20kg were evaluated. Among the small-sized dogs, the included breeds were: Shih Tzu (3), Pomeranians (2), Pug (1), and two mixed-breed dogs. The medium/large-sized dogs included one Boxer, one Border Collie, two American Pit Bull Terriers, one Great Dane, and one mixed-breed dog. For group 2, there were evaluated six dogs with >20kg were evaluated, including one German Shepherd, one Belgian Malinois, one Labrador Retriever, one American Pit Bull Terrier, and two mixed-breed dogs. Group 3 comprised five dogs weighing >15kg, including one Dobermann, one British Pointer, one Labrador Retriever, and two mixed-breed dogs.

There was a tendency for the average pixel count to be influenced by body weight (P= 0.0774), although this effect did not reach statistical significance. Neither testicular side (right or left) nor age group influenced the average pixel count in the present study.

**Figure 2:**
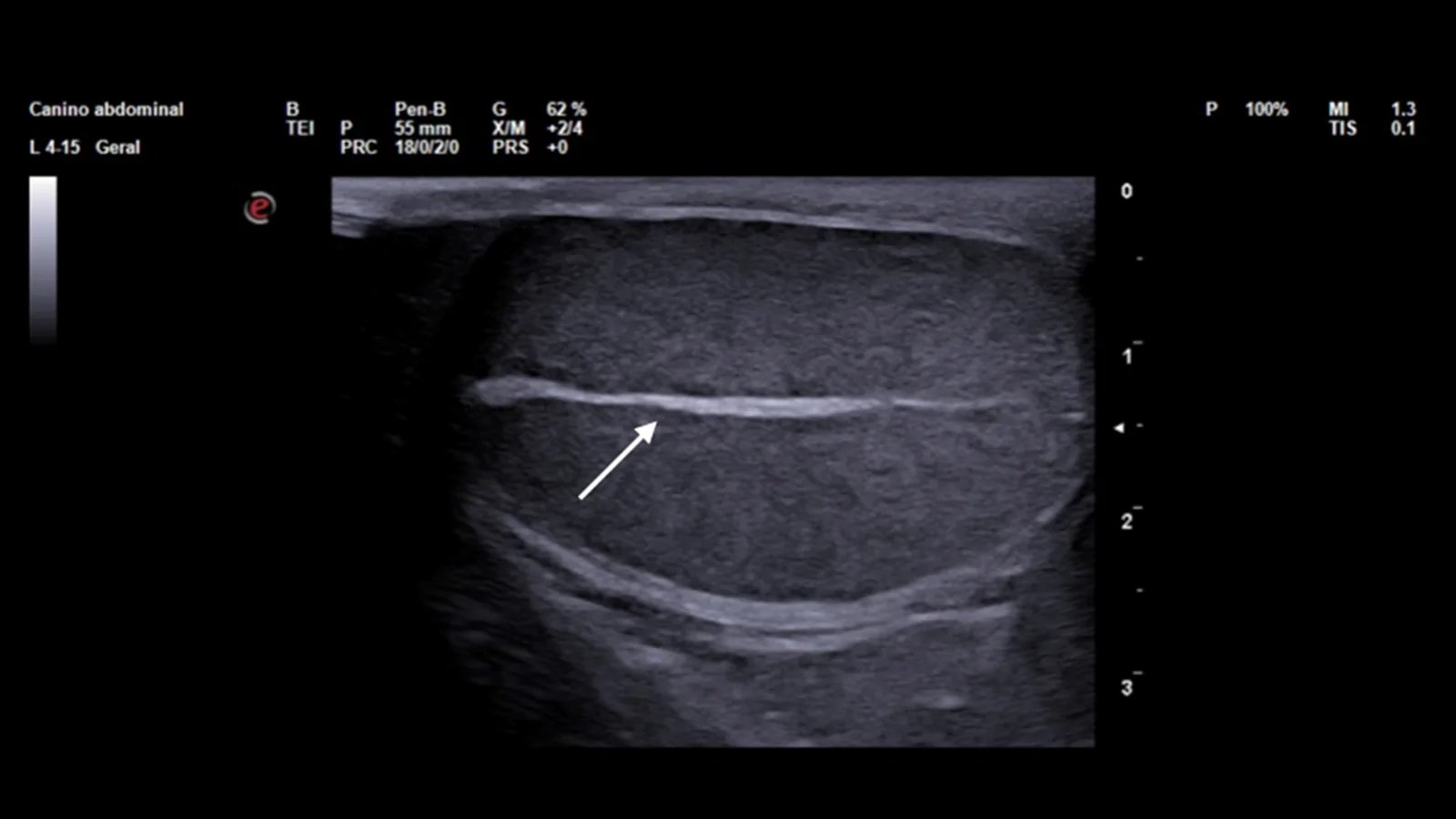
B-mode ultrasonographic image of normal canine testis, appearing as an oval, hypoechoic and homogeneous structure with regular, well-defined contours and a central mediastinal line (arrow).

During B-mode evaluation, the testes appeared as oval, hypoechoic structures with a central hyperechoic mediastinal line, homogeneous echotexture, and regular, well-defined contours (Figure 2).

**Figure 3:**
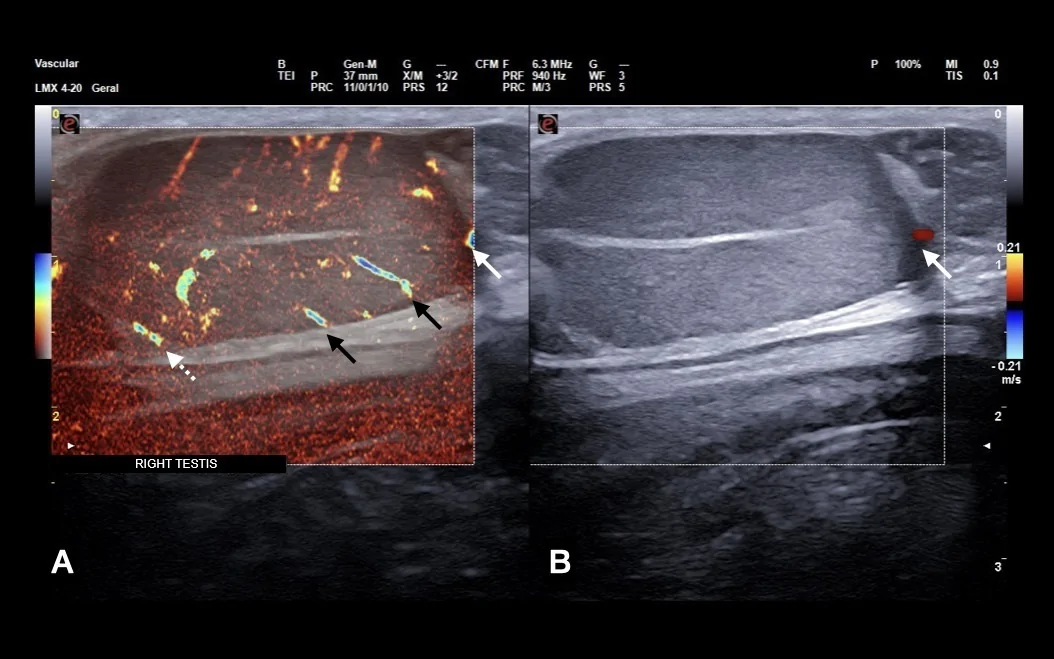
Testicular vascularization in the canine testis evaluated by MicroV (A) and color Doppler ultrasonography (B), highlighting the intratesticular arteries (dashed white and black arrows) and marginal arteries (white arrows).

Color Doppler imaging most clearly demonstrated the testicular artery branches in the intratesticular region, with scattered color signals in the marginal region. MicroV showed vascularization in all parts of the organ, not only in the form of dots and fragments, but also revealed the branched structure of microvessels (Figure 3).

**Figure 4:**
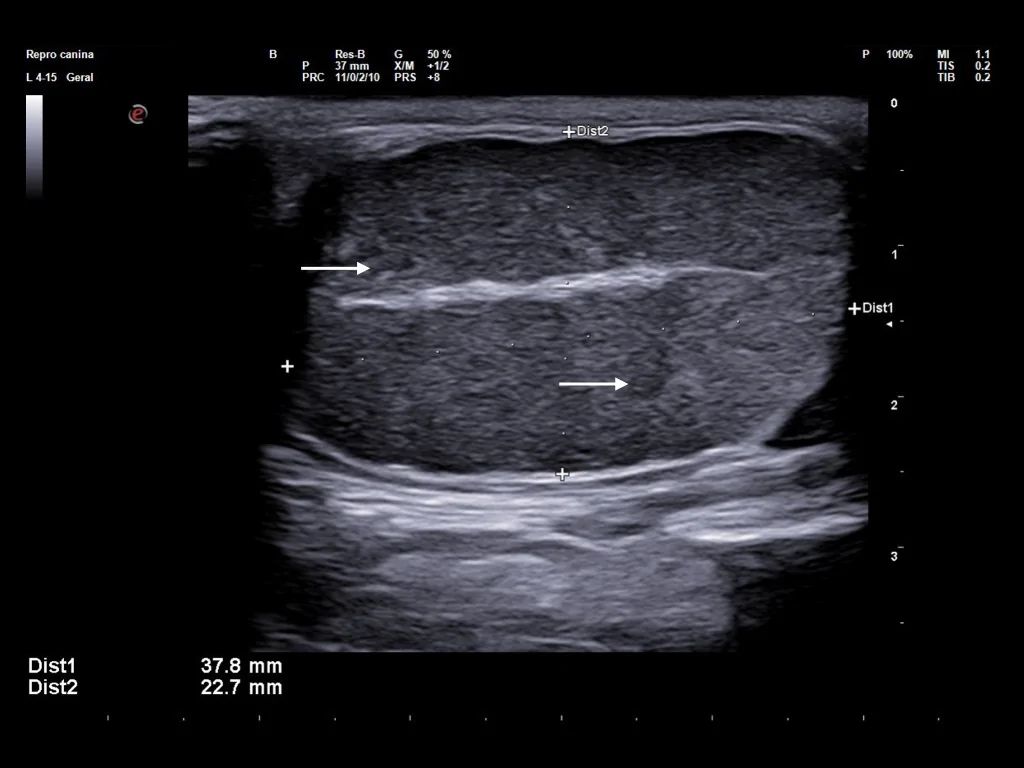
B-mode ultrasonographic image of a canine testis showing inhomogeneous parenchyma, characterized by diffusely distributed punctate anechoic areas (white arrows).

In approximately 44% (11/25) of dogs, regardless of age group (five from G1, three from G2, and three from G3), inhomogeneous echotexture of varying intensities was observed, with the presence of diffuse anechoic areas (Figure 4), without association with alterations in vascularization on MicroV and color Doppler. Of the 11 dogs with inhomogeneous testicular parenchyma, eight dogs weighed >25kg (ranging from 26 to 50kg) and belonged to the breeds German Shepherd, Labrador Retriever, American Pit Bull Terrier, English Pointer, and Mixed. Three dogs weighed <5kg (ranging from 3 to 4kg) and belonged to the breeds Shih Tzu and Pomeranian.

**Figure 5:**
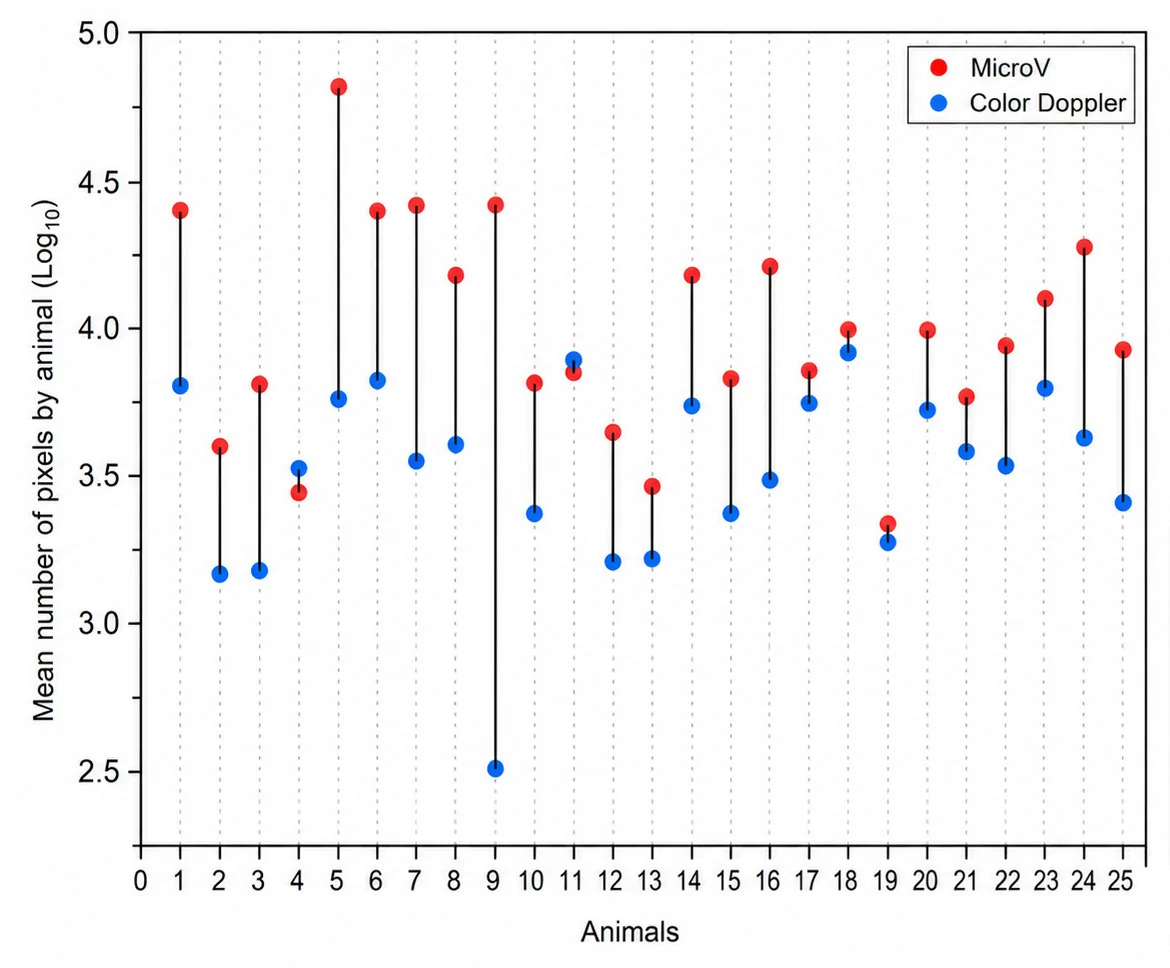
Paired dot plot representing the mean number of pixels obtained with Color Doppler (blue dots) and MicroV (red dots) for each one of the 25 dogs in the study. Values are presented on the log-link scale.

The evaluation of vascularization using the MicroV technique, compared with color Doppler, showed a significant increase in the number of blood vessels, estimated by subjective visual assessment (Figure 1 and 3). Those qualitative observations were confirmed by a significant increase in the number (Figure 5) and intensity of pixels, objectively quantified by the ImageJ digital image analysis software.

In the statistical analysis, the Type III test for fixed effects showed that the mean number of pixels differed significantly between Doppler and MicroV (P< 0.0001) (Table 2). The estimated mean pixel count was approximately 2.5-fold higher for MicroV than for color Doppler (Table 3).

**Table 2:** Analysis of each effect on pixel quantification using Type III tests of fixed effects.

**Effect**	**Num DF**	**Den DF**	**F value**	**Pr > F**
Imaging modality	1	67	67.71	<0.0001*
Testicle	1	67	0.11	0.7444
Imaging modality*testicle	1	67	0.01	0.9072
Age group	2	67	0.32	0.7245
Body weight	1	67	3.22	0.0774*

* Statistical significance (or trend) considering a significance level of 5%.

**Table 3:** Effect of the imaging modality on pixel quantification analyzed by Least Squares Means.

**Imaging modality**	**Estimate**	**Standard Error**	**DF**	**t Value**	**Pr > t**	**Mean**	**Standard Error**
							**Mean**
Doppler	8.1680	0.1300	67	62.81	<0.0001	3526.24*	458.59
MicroV	9.0903	0.1300	67	69.91	<0.0001	8868.46*	1153.20

* Represents mean values of pixels, presented on the original scale (i.e., number of pixels) for Doppler and MicroV. Estimated values are presented in the log-link scale.

## Discussion

In the present study, satisfactory-quality ultrasonographic images were successfully acquired without trichotomy of the testicular region, corroborating the literature (Kinns & Nelson, 2019). A high-frequency linear transducer (7.5 MHz) is the preferred option for the examination that should be performed with the scrotum stabilized by the operator’s hands or by an available assistant (Kinns & Nelson, 2019). Ultrasound evaluation performed with a microconvex transducer was also suitable for testicular evaluation, although the images obtained with a linear transducer have higher resolution, favoring the evaluation of vascular and, above all, microvascular structures.

The ultrasound characteristics of the testicular parenchyma in B-mode were consistent with the descriptions in the literature (Lemos et al., 2020; Souza et al., 2017; Venianaki et al., 2023), showing a homogeneous, well-defined structure with an echogenic mediastinal line.

In this study, some testes showed areas of slight irregularity, classified as inhomogeneous regions. The heterogeneity of the testicular parenchyma has been linked to sperm production in previous studies, becoming evident with the onset of fertility and increasing with age in dogs (Moxon et al., 2015; Venianaki et al., 2024). Conversely, some studies have reported that the presence of testes with heterogeneous echotexture was associated with low sperm quality (Souza et al., 2017). However, no andrological assessments were performed in this study that could be correlated with the above-mentioned ultrasound findings and fertility.

These inhomogeneous regions may be considered an incidental finding of the present study, likely associated with the increased anatomical detail provided by high-resolution ultrasonography, and warrant further investigation. Notably, 8 of the 11 dogs exhibiting inhomogeneous testicular parenchyma were large-breed dogs. Although the clinical significance of this observation remains unclear, larger testicles may allow more detailed ultrasonographic assessment, thereby increasing the likelihood of detecting subtle parenchymal heterogeneity that may go unnoticed in smaller individuals.

Breed predisposition has been reported for several canine reproductive disorders. A high detection rate of testicular tumors was identified in mixed-breed dogs, Yorkshire Terriers, Labradors, Golden Retrievers, and Fox Terriers (Gazin et al., 2022). Other authors reported a predisposition of Shetland Sheepdog, Boxer, German Shepherd, and Afghan Hound for testicular tumors (Liao et al., 2009). Poodle and Pinscher breeds showed higher risks for general testicular disorders (such as inflammatory, degenerative, and neoplastic) (Andrade et al., 2026). However, the association between breed and subtle testicular parenchymal alterations was not assessed in the present study and would require further investigation using larger homogeneous samples.

Another possible explanation is that these findings represent mild structural alterations associated with age-related testicular degeneration. Previous research reported that dogs aged >7 years presented a higher prevalence of testicular disorders (Andrade et al., 2026). However, this hypothesis is not strongly supported by the present data, as no apparent age predisposition was observed among the dogs exhibiting such alterations. Furthermore, histopathological evaluation was not performed. For that reason, further investigation through studies incorporating histopathological correlation is important to establish the nature and clinical significance of these ultrasonographic findings.

The heterogeneity of the testicular parenchyma should be further quantified with an objective approach, based on measuring the number of pixels and variation in their intensity across different regions of the testicular parenchyma, as described previously (Moxon et al., 2015). However, the present study aimed to analyze pixel data to quantify vascular structures; the investigation of testicular parenchyma heterogeneity and the evaluation of testicles with pathological conditions were not the focus of the present analysis and may be investigated in future studies.

A trend was observed that the number of pixels detected by the Doppler and MicroV techniques varied with dog weight. A possible association with vascular anatomy may be suggested, as vascular patterns may vary with animal size (Souza & Silva, 2014). These authors reported that testicular Doppler flowmetry values varied with dog size, with higher velocity vessels found in the marginal region in small dogs and in the spermatic cord region in large dogs. Additionally, larger dogs had a larger testicular artery diameter (Colatti et al., 2020).

Regarding the age of the animals, the literature indicates that aging is associated with decreased testicular vascularization in dogs (Brito et al., 2021). Thus, it would be expected that in older dogs, the MicroV technique would be more effective than color Doppler for vascular investigation. However, the present study did not demonstrate a statistically significant difference between the techniques, possibly due to the sample size.

Testicular Doppler ultrasonography allows visualization of the testicular artery, which is most readily identified in the supratesticular region of the spermatic cord, although it can also be evaluated within the marginal and intratesticular portions of the testis (Carrillo et al., 2012). In this study, testicular evaluation with color Doppler allowed the detection of evident supratesticular vessels. Characterization of intratesticular vessels was possible in some but not all of the testes evaluated. Besides the main testicular artery, the testes are organs richly irrigated by low-caliber vessels and blood flow (Visalli et al., 2024). These could not be evaluated by this imaging modality in the present study.

The increase in the number of blood vessels and pixels obtained using the MicroV technique, compared to color Doppler, demonstrated the greater detection capacity of this technique for evaluating testicular microvascularization. These findings corroborate studies with adult human subjects (Durmaz & Sivri, 2001), children (Karaca et al., 2016), and neonates (Ayaz et al., 2019), in which the MicroV technique identified testicular vascularization in 98% of cases, when compared to color Doppler (only 70% of cases) (Visalli et al., 2024).

Furthermore, this modality showed a more evident and diffusely positioned vascular network, characterizing its greater spatial distribution when compared to color Doppler, which detected marginal vessels and few portions of larger intratesticular vessels. These findings are consistent with descriptions in the literature on testicular vascularization in humans, in which color Doppler detected intratesticular arteries as small fragments or colored dots, which may be confused with artifacts, especially before puberty (Visalli et al., 2024). Comparatively, the same authors reported that the MicroV technique provided a more detailed assessment and greater conspicuity of the testicular vascular network when compared to color Doppler in both young and adult patients. In veterinary medicine, microvascular imaging reportedly produced better visualization and resolution of renal microvascular structures in dogs when compared to the B-mode and Color Doppler assessments (Koh et al., 2025)

The present study did not evaluate animals with testicular pathologies; however, the potential of MicroV for investigating testicular disorders should be considered. Most testicular disorders involve inflammatory and neoplastic processes, which are associated with increased volume and vascularization, or ischemic processes (as in physiological aging). Therefore, rapid assessment of microvascular flow may be important and may be better achieved using these novel ultrasonographic techniques, potentially enabling early diagnosis of testicular conditions before detectable changes in parenchymal echogenicity and echotexture occur (Brito et al., 2021).

However, the MicroV technique is not capable of showing the direction of flow (Visalli et al., 2024) or providing a quantitative assessment, and its efficiency is compromised when the target structure is located at a greater depth. Other important points that should be considered are the need for specific imaging technologies, which may not be widely available in all locations, and the interference that can still occur from adjacent movements, resulting in noise artifacts, which must be differentiated from blood flow by visualizing the pulsation in it (Aziz et al., 2022). Thus, the importance of a multimodal approach combining Doppler and MicroV assessments should be emphasized to leverage the benefits and contributions of each technique.

This study had some limitations, as the sample groups were heterogeneous and differed in the number of animals. Regarding imaging acquisition, the gain and other imaging parameters were individually optimized for each patient based on patient size and image quality requirements. This may represent a source of operator-dependent variability. Despite the use of a consistent processing approach and single-blinded operator evaluation to minimize variability in the pixel quantification, this semi-manual approach may also represent a limitation of the methodology.

For future studies, better control and definition of ultrasound technique adjustments, along with a larger sample size and a more homogeneous distribution of individuals within each experimental group, may provide greater reproducibility and more robust statistical results.

The results support the proposed hypothesis, showing greater sensitivity and resolution of the MicroV technique in the evaluation of testicular vascularization. Additional studies including canine patients with testicular disorders are important to determine the potential of this imaging modality to improve the diagnostic accuracy of testicular diseases.

## Conclusion

The analysis of the findings enabled detailed observation and characterization of testicular vascularization and microvascularization in dogs, highlighting the microvascular flow (MicroV) ultrasound imaging technique as a promising complementary method for canine andrology and small animal clinical medicine in general. MicroV stands out for providing detailed information on low-flow and low-velocity vascular pathways that are not readily captured by conventional Doppler methods.

In conclusion, MicroV enabled a more detailed assessment of testicular vascularization compared with conventional color Doppler in the dogs evaluated in this study. Although the findings are promising, further studies involving a larger and homogeneous population and including testicular pathological conditions are necessary to better establish the diagnostic applicability and clinical relevance of this technique for testicular investigation in dogs.
